# NEVA: Visual Analytics to Identify Fraudulent Networks

**DOI:** 10.1111/cgf.14042

**Published:** 2020-06-19

**Authors:** Roger A. Leite, Theresia Gschwandtner, Silvia Miksch, Erich Gstrein, Johannes Kuntner

**Affiliations:** ^1^ Faculty of Informatics Vienna University of Technology (TU Wien) Vienna Austria; ^2^ s IT Solutions AT Spardat GmbH Vienna Austria; ^3^ Erste Group IT International Wien Austria

**Keywords:** visualization, visual analytics, financial fraud detection

## Abstract

Trust‐ability, reputation, security and quality are the main concerns for public and private financial institutions. To detect fraudulent behaviour, several techniques are applied pursuing different goals. For well‐defined problems, analytical methods are applicable to examine the history of customer transactions. However, fraudulent behaviour is constantly changing, which results in ill‐defined problems. Furthermore, analysing the behaviour of individual customers is not sufficient to detect more complex structures such as networks of fraudulent actors. We propose NEVA (Network dEtection with Visual Analytics), a Visual Analytics exploration environment to support the analysis of customer networks in order to reduce false‐negative and false‐positive alarms of frauds. Multiple coordinated views allow for exploring complex relations and dependencies of the data. A guidance‐enriched component for network pattern generation, detection and filtering support exploring and analysing the relationships of nodes on different levels of complexity. In six expert interviews, we illustrate the applicability and usability of NEVA.

## Graphical abstract

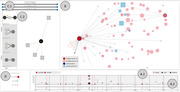

## 1 Introduction

Detecting and understanding the network of related events is an important task in several domains such as biology, medicine, insurance companies, retail companies, public sector and banks. Consider the following sample questions: ‘How are those diseases linked?’, ‘What is the influence of this component on that treatment?’, ‘Which selling products are linked, how and why?’ (see ‘the parable of the beer and diapers’ case [Whi11]).

In financial institutions, network analysis is used to understand users' behaviour and to detect frauds. They often use automatic algorithms to detect fraudulent events of single actors, such as assessing if a transaction fits the transaction history of the respective customer. The goal of such a technique is to reduce the amount of false‐positive and false‐negative findings, to avoid harm in a magnitude of hundreds of thousands of dollars. However, this type of individual analysis system is sensitive and needs to be in constant evaluation in order to ensure the quality of the results.

In this work, we focus on the detection and analysis of fraudulent networks of bank transaction events. We analyse visual patterns for different types of frauds (i.e. unauthorized transactions, money laundering and straw persons). Besides dealing with a data type with complex features such as time‐oriented and multi‐variate aspects [AMST11], we propose the exploration and reasoning about a network of individuals.

Pattern and outlier detection are common tasks of Artificial Intelligence (AI) approaches in financial fraud detection (FFD).

The main challenge of applying AI techniques to FFD is the constant adaptions required due to the creativity and fast change in fraudulent strategies which lead to false‐negative findings. False‐positive and false‐negative findings come at expensive costs. While false‐positive alarms might lead to the accusation of innocent people, false‐negatives mean that fraudsters succeed to cause financial harm to the bank or to innocent bank customers. While these are two different circumstances, the fine‐tuning of detection algorithms for both need to be done in combination. More sensitive algorithms lead to the detection of more frauds but also generate more false‐positive alarms. On the other hand, a more generic algorithm would detect fewer frauds, and thus, more false‐negative alarms would be generated. Consequently, the calibration of their sensitivity is essential. Thus, in order to improve FFD, it is important (1) to constantly adapt and balance the parameters of algorithms to identify as many fraudulent cases as possible without triggering too many false alarms and (2) to support informed reasoning about the output of these algorithms, such as potential false‐positive and false‐negative results.

Types of financial fraud are classified with respect to different features, such as the amount of money, time of the transaction and the relations of involved accounts. Besides applying AI for pattern and outlier detection, one of the main challenges on FFD is detecting fraudulent networks. However, performing subgraph search is an extremely demanding task and it is characterized as an NP‐complete problem. Even with punctual optimization available [Nei12, SSMIN16], this task would require a sweep of all nodes and their relation layers recursively. Hence, this results in costly algorithms. Since the negative impact of fraudulent attacks increases over time, the task of FFD is of social and financial importance and it must be detected as fast as possible.

Tasks of fraud detection are an open problem that require visual exploration, discovery and analysis [KTM09]. Visual Analytics (VA) would offer great benefits by integrating human analysis into this complex process [KMS*08]. However, the current solutions for FFD mainly use data mining techniques, with just a few exceptions involving VA techniques. Thus, we propose a VA approach for the investigation of fraudulent networks, based on an automatic FFD alert system. In this work, we focus on detecting specific types of financial frauds, such as ‘straw persons’ and ‘money laundering’ within a financial institution. We designed this approach with respect to Munzner's nested model [Mun09], which makes it flexible and extensible enough to be adapted to similar domains with similar multi‐variate, relational and time‐oriented aspects. We also used the ‘Design Triangle’ by Miksch and Aigner [MA14] to identify the target audience, define the data model and find the important tasks in the target domain. Thus, our main contributions are:

- Designed in close collaboration with domain experts, NEVA (Network dEtection with Visual Analytics) improves the network analysis for FFD by intertwining automatic methods and visualization techniques within an interactive exploration environment (Section 4).
- To the best of our knowledge, we present a new guidance‐enriched component for network pattern generation, detection and filtering that supports different levels of analysis complexity (Section 4.4).
- We illustrate the applicability and usefulness of NEVA with four real‐world tasks and discuss the lessons learned from an evaluation session with six domain experts (Sections 5 and 6).
- We identify and elaborate on open challenges and possible future research directions in the field (Section 7).

## 2 Related Work

This work is mainly motivated by the same problem discussed in [LGM*18]. However, a network exploration and analysis perspective is brought by NEVA, to better contextualize that we categorize related works into the following seven topics:

##### VA for the financial domain

Looking for visual approaches for financial data, we identified FinanceVis [DML14] which is a survey that provides a browser tool that includes over 85 articles related to financial data visualization. Moreover, Ko *et al*. [KCA*16] presented a survey of approaches for exploring financial data. Motivated by a lack of information available, financial data experts were interviewed about their preferences regarding automated techniques, visualizations, data sources and interaction methods. This survey highlights the many underexamined financial business domains and argues for the need of more works presenting design, development and results involving real‐world financial data, as our approach does.

##### Areas of Fraud detection

There are several state‐of‐the‐art reports with emphasis on general fraud detection. One of the first modern analysis approaches concerning fraud detection was published by Bolton and Hand [RJB02] in 2002. They identified four areas: credit card fraud, money laundering, telecommunication fraud and computer intrusion. The same types of fraud were described by Kou *et al*. [KLSH04] but are broadly classified into: misuse and anomaly detection. These works supported our understanding of the diverse fraud domains and their common approaches. However, no analysed work presented a multi‐variate, time‐oriented, network VA approach for FFD of banks transactions.

##### Financial fraud detection

One of the pioneers of designing visual solutions to support FFD analysis was Kirkland *et al*. [KSH*99]. They present a tool called NASD, which uses visual techniques to facilitate the interpretation of detected frauds. NASD's regulation Advanced‐Detection System (ADS) is supported by five different visualizations. Moreover, ADS combines detection and discovery features that can support different domains. This work presents AI for pattern recognition, visualizations to aid human reasoning and data mining to support regulatory analysis. While this was pioneer work in the field of FFD, they do not live up to state‐of‐the‐art features such as interactive exploration of the visualized data. Argyriou *et al*. [ASV14] aimed to find fraudulent activities committed by employees of a company (internal frauds). In contrast to that, we aim to investigate other types of frauds: fraudulent schemes involving a network of users (i.e. money laundering schemes).

Another approach for FFD is presented by WireVis's [CGK*07], using multiple coordinated views. The main idea is to explore big amounts of transaction data through interactive views in order to aid fraud detection. The approach highlights similarities between accounts based on transaction keywords over time. WireVis clusters a set of accounts based on their similar keywords, depicting relationships among accounts and keywords over time. Thus, this approach is limited to analysing transactions that present similar keywords in its description. Due to the similar data type and the use of multiple connected (and interactive) views, this is the most similar approach to NEVA. However, NEVA is especially focused on the detection and analysis of fraudulent networks, and thus, provides specialized means for network pattern analysis.

Our previous approach, ‘complementing work' [LGM*18], tackles the problem from a different direction and presents an integration of VA methods into an existing ‘detection and decision’ workflow. This approach combines automatic methods with well‐known visualization techniques in order to lower the learning effort for domain experts. We developed our approach following the same ‘familiar well‐known visualization’ design thinking. Different from EVA [LGM*18], NEVA aims FFD support to frauds investigations involving network aspects such as ‘money laundering’ and ‘straw person’. On the other hand, EVA [LGM*18]'s main goal was the discovery and reasoning about frauds coming from individuals history analysis as ‘unauthorized transactions’.

##### Automatic methods for FFD

A first financial flow analysis approach is presented by [SK95]. This work focuses on data aggregation in order to allow users to draw analytical conclusions and make stock decisions. A more modern approach, EventFlow [MLL*13] is a query and data transformation tool for temporal event data sets designed to facilitate analysis. This approach provides aggregated data visualization representations to track events that are related over time.

Recent needs in FFD are presented by Dilla and Raschke [DR15]. The authors presented theoretical framework to predict how the investigators should apply VA techniques. They evaluated various visualization techniques according to different cognitive processes. The discussion about the benefits of interactive data visualization for fraud detection was one of the main discussion topics, which was also used as one of the main points of our research. A decision support based on profile generation and analysis is presented for online banking fraud analysis from Carminati *et al*. [CCM*14]. This semi‐supervised approach provides no visual support for fraud analysis. However, it is directly related to our approach since we are also focusing on profile analysis for fraud detection. This approach has a strong statistical meaning. We believe that VA methods have great potential to improve the investigation of the FFD and enable the analyst to better understand the lacks of the scoring systems.

##### Networks in finance

Allen and Babus [AB09] presented a non‐visual paper that discusses a node–link representation as being a natural financial system representation which can efficiently explain certain economic phenomena. Tekušová and Kohlhammer [TK08] presents a node–link diagram that is generated using economic analysis methods. The system aims at showing patterns for large corporate shareholder networks. It allows the visual analysis of cash flows and the identification of shareholders. When targeting event monitoring works, Huang *et al*. [HLN09] presented a VA framework for stock market security. Aiming for a reduction of false alarms produced by traditional AI techniques, this work presents a visualization approach combining a node–link diagram for network analysis and a 3D treemap for market performance analysis. Didimo *et al*. [DLM14] presents a VA tool that allows the analysis of different institutions and also the analysis of internal transactions of a bank is considered. The paper describes the two different types of network to correctly model the data.

One of the biggest influence to develop our approach comes from the work of Cheng *et al*. [CNY*17]. This VA approach to loan network risk management presents risk measurements by analysing subgraphs flows in a bigger network. In this work, 20 subgraphs are analysed during the study case. Even subgraph search being an NP‐complete problem there are algorithms that try to optimize the performance of the task [SSMIN16, ZC16]. These subgraphs models coupled with the subgraph search challenge inspired us to create a ‘Node‐Link Patterns’ concept that allows us a subgraph exploration with guidance (see Section 4). Wang *et al*. [WLS*18] proposed a system of multiple coordinated views that support anomaly detection for global trade network analysis. The system takes localization and events (i.e. armed conflicts) into consideration and relates them to international trades.

##### Graph query

Some works have been demonstrating the efficiency of using visual language to filter graphs in order to find expected patterns and results [PHT*17, PTE*16]. On the other hand, VIGOR [PHE*18] is focused on supporting users to better reason about graph query results (grouping nodes, adding labels to clusters and so on). Parts of our approach allow also a ‘drawable’ query design similar to [KZA10, CFT*08]. TeFNet aims to contrast tax evasion, money laundering and fiscal frauds [DGL*19]. The query language and the visualization techniques rely on a suitable timeline approach that maps time to space. The approach presents efficient results for large graphs. Like NEVA, the system has been tested in a real working environment. However, NEVA provides means for ‘drawable’ queries (see Section 4.4) developed especially for FFD. Moreover, different from the auto‐completion guidance during query writing implemented by VISAGE [PTE*16], NEVA adds guidance support during pattern drawing/querying (see Section 4.4). After discussing with FFD experts, we decided to exclude approximate results as GRAPHITEs [KZA10] does only precise results in order to not mislead any search or pattern understanding.

**Guidance** started in the area of Human Computer Interaction (HCI) [Hor99] and was recently characterized in the context of VA by Ceneda *et al*. [CGM*17]. Ceneda et al. define: ‘Guidance is a computer‐assisted process that aims to actively resolve a knowledge gap encountered by users during an interactive visual analytics session’. The authors extend van Wijk's model of visualization, and thus, they present a general model to facilitate in‐depth reasoning about guidance. To illustrate their model with examples, they use existing guidance approaches from the literature. Three distinct degrees of guidance are described by Ceneda *et al*.: (1) Orienting, techniques that support building and preserving a mental map, (2) Directing, presents to the user a pre‐selection pool of possibilities (a recommendation system is an example) and (3) Prescribing, techniques that force the user to take the recommended next step. In a literature review about guidance in visual data analysis [CGM19], the authors highlighted the different guidance degrees (Prescribing, Directing and Orienting) which can be used to classify guidance. The guidance‐enriched features implemented in NEVA, present a degree of Directing guidance because they provide a set of alternative options that guide the analyst to avoid the formulation of useless queries (see Section 4.4).

## 3 Financial Fraud Detection

We designed, developed and evaluated our approach in collaboration with a national bank [Ers18]. Our main goal was to improve the current FFD techniques used by our partner institution. However, before we present NEVA, we briefly discuss (1) the characteristics of transaction data, (2) the complexity of detecting networks of fraudsters, (3) a summary of the currently used pipeline for FFD and (4) the scoring approach used to identify suspicious transactions. Since we give a detailed description of these aspects in one of our previous works [LGM*18], we just summarize the main aspects here in order to ground our contribution.

### 3.1 Transaction data

We used real data of money transactions from our bank partner for the development of our approach. The data from a 2‐year period that represents payments and money transfers were anonymized and internally checked by the bank before they made it available to us. The transaction events have several categorical, numerical, geospatial and temporal dimensions (i.e. money sender/receiver, amount of money, location, time of execution, and others). While our approach is focused on investigating relational features, we also support the exploration of the most critical data dimensions (such as the amount of money) that were identified by our collaborating domain experts. Moreover, we defined different categories of node types such as money senders and receivers based on Cheng *et al*. [CNY*17] (see Section 4.4).

### 3.2 Problem complexity

Fraudsters tend to adapt their fraudulent strategies continuously. This is why automatic algorithms, for instance, searching for a pre‐defined set of patterns, is unlikely to succeed in the context of FFD. Automatic means can be used to identify abnormalities, however, a human investigation is required to confirm the harmfulness of suspicious cases. To support this task, VA techniques enable the human to interact with the data and to improve the reasoning process. In addition to frequent pattern changes of fraudulent attempts, several other aspects add to the task complexity of FFD:

In one of our previous works [LGM*18], where we approach a related problem of FFD and investigate data with the same characteristics, we go into details about: (1) the scalability complexity of dealing with hundreds of thousands of transactions per day, (2) the context complexity of understanding the motivation behind a financial crime, (3) the complexity of time‐oriented data analysis [AMST11] that might obfuscate some frauds and (4) the problem of fraud classification which might include a huge number of subclasses of frauds due to the multi‐variate nature of the data. All these aspects were addressed during NEVA‘s design and implementation.

### 3.3 Methodology for FFD

In this subsection, we give an overview of the workflow pursued by our collaborators to identify and reason about financial frauds. According to the privacy policy of our collaborating bank, we cannot go into details about the actual fraud detection algorithm. However, we roughly sketch the four steps of the used methodology: profile generation, score generation, results interpretation and fraud validation.

3.3.1

##### Profile Generation

**Figure 1:**
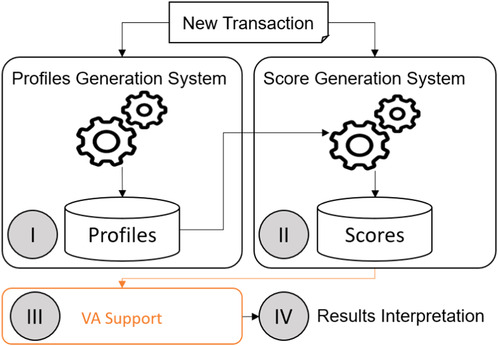
The design overview of the transaction evaluation system. We present a new interactive VA approach to support the investigation of suspicious behaviour. We highlight in orange how our approach fits into the workflow of FFD (III).

For each customer, an automatic system generates a profile based on the transaction history of his/her account (see Figure 1(I)). Profile generation is a process that has its own rules of execution and it is not synchronized with the other steps of the workflow (i.e. profiles are recomputed every week or every 10 transactions).

##### Score Generation

Each incoming transaction is compared with the respective customer's profile (see Figure 1(II)). For a new transaction, the algorithm considers data dimensions such as: operation location, operation time, amount of money and other features to compare the new action with the usual behaviour of that customer (i.e. the customer's profile). Then, the algorithm generate subscores for each dimension and summarize them into one overall score. The higher the score, the more suspicious is the transaction.

##### Results Interpretation

This is the non‐automatic phase of the investigation where the investigators analyse multiple transactions simultaneously, due to time constraints. Transactions whose scores are over a given threshold are further filtered by pre‐defined rules. Based on their personal experience, the investigators then decide whether an alarm should be considered fraudulent or not (see Figure 1(IV)).

##### Fraud Validation

After deciding for a suspicious transaction, a further personal investigation is required. The bank might stop the transaction in some cases. From this step on, there are different legal approaches that might be applied according to the type of fraud that it seems to be.

### 3.4 Types of frauds

While types of frauds are manifold, in this subsection, we briefly describe the types of frauds that we focus on.

Money Laundering aims to transform money from crime and corruption into legal money. Mostly, this type of fraud involves a network of accounts. Straw Persons main goal is using someone with low suspiciousness levels to get illegal money for someone else who is not legally allowed to receive it. Unauthorized Transactions involves transactions that were sent from a customer account but were not authorized by the account owner.

### 3.5 Scoring approach

Due to security and privacy constraints of our collaboration partners from the bank, we cannot describe the scoring algorithm in detail. However, we compare it to another public approach from Carminati *et al*. [CCM*14]. In this approach, customer profiles are generated in a semi‐supervised way outputting several kinds of statistical measurements. This approach flags transactions as suspicious. When evaluating our scoring algorithm by comparing it to the old scoring approach used by the bank, we could confirm that domain experts could detect 500% more confirmed fraudulent transactions, preventing 86% of the financial losses [LGM*18].

## 4 NEVA Design and Implementation

We design NEVA by using a combination of two methodologies: ‘Nested Model' [Mun09], and ‘Design Triangle' [MA14]. While the nested model methodology guarantees a flexible and extensible interactive approach, the design triangle methodology supports a better understanding of the scope of the problem by the clarification of the Data, User and Tasks.

4.1

4.1.1

##### Data

Financial transaction events constitute multi‐variate, relational and time‐oriented data, including details about the transactions such as amount, time, sender, receiver, etc.

##### Users

Investigators from financial institutions that investigate and validate alerts of suspicious transactions.

##### Tasks

The overall tasks are network‐related fraud detection and reasoning by means of profile analysis. These tasks include mainly the reduction of false‐negative and false‐positive alarms, history comparison, as well as the manual investigation of suspicious transactions.

### 4.2 Requirements

In the context of fraud detection and the analysis of financial networks, there are still many unresolved challenges [KLSH04, AB09]. With our VA solution, we focus on supporting the tasks of ‘results interpretation’ and ‘fraud validation’ (see Section 3.3). To support fraud investigators during a better decision‐making process, in collaboration with the domain experts, we agreed to address the following requirements:

4.2.1

##### R1: Identification of False‐Negative Alarms

Aiming to overcome the lack of accuracy of automatic algorithms, the exploration and identification of hidden fraudulent accounts is an important task that should be supported by the proposed solution. The system needs to facilitate the reasoning about potential criminal networks. It should not only be possible to investigate suspicious transactions but also the accounts linked to them. By visualizing non‐flagged accounts (i.e. accounts that were not flagged as suspicious by the automatic detection mechanism) in relation to flagged accounts (i.e. possible fraudulent accounts), investigators might be pointed to suspicious behaviour that could not be detected by automatic algorithms.

##### R2: Identification of False‐Positive Alarms

False‐positive alarms are transactions that were flagged by the automatic system as potential frauds but actually they are not. The amount of false‐positive alarms varies according to the calibration of the automatic system. However, 100% precision cannot be achieved (i.e. a good trade‐off has to be found between the number of false‐positive and false‐negative alarms), and thus, there will always be false‐positives. Flagged transactions, however, lead to the investigation of the involved accounts and their related network. This group of accounts need to be inspected in order to reason about the potential fraud, which leads to a further decrease of false‐positive alarms. To support informed decision making during the validation step (see Section 3.3), the system has to support the visual analysis of the network of flagged accounts and its related network.

##### R3: Identification of Different Types of Frauds

With the current system, it is possible to automatically identify unusual behaviour. However, the classification of this behaviour is still an open issue. Classification, however, is essential to better understand the possible consequences of an alarm and make better informed decisions on how to handle it. The system should support the investigation of suspicious behaviour of accounts that are involved in flagged transactions and their related network. Thus, based on the money flow of accounts, the identification of different types of frauds should be possible.

##### R4: Guided Network Pattern Exploration and Search

The changing behaviour of fraudulent attempts needs to be constantly monitored. Thus, our system needs to provide efficient support to reason about identified patterns of possible fraudulent attempts. Moreover, searching for specific types of frauds (i.e. patterns) within the comprehensive network of financial transactions should be supported. However, searching for arbitrary patterns within a huge network of financial transactions might easily lead to cognitive overload. Thus, the user needs guidance on which patterns are worth investigating.

### 4.3 Data setup

Aiming to improve the current methodology for FFD (see Section 3.3), we kept our main focus on analysing suspicious transactions. Thus, instead of displaying the 77,000 accounts at once, we propose an automatic initial filter based on the investigators’ workflow. Thus, we suggest a step‐wise approach to identify additional fraudulent cases that could not be identified by automatic means. First, (1) we apply a filter on the data set to select just accounts that contain alarming transactions. Next, (2) we select accounts that had relations to at least two suspicious accounts. This process is called ‘man‐in‐the‐middle’ selection (see an example of a ‘man‐in‐the‐middle’ in Figure 8). Lastly, (3) we analyse the relations of all found ‘man‐in‐the‐middle’ accounts that are not only receptors but also senders of money in order to find newly hidden ‘man‐in‐the‐middle’ accounts (this case is represented in Figure 7).

While the majority of accounts selected in this phase might not seem suspicious at first sight, the investigators consider that ‘man‐in‐the‐middle’ accounts represent a certain logistic risk and require further investigation. Those accounts are likely to be part of fraudulent schemes. This analysis was previously conducted by analysing spreadsheets. So, the data presentation made by our VA approach in addition to the automatic FFD mechanism enhances the possible analysis scope by allowing investigators to interact visually with the data.

### 4.4 Network event detection with visual analytics

The main idea of our proposed approach started as a further work proposed by [LGM*18]. As a sequel to our former work, we again developed this approach following the iterative design process [Mun09]. We had several meetings with our collaborators to discuss the data, the context, the problem and the tasks. During the design process, we created a number of visual encoding designs and asked for expert feedback. We iteratively designed several prototypes with which we could test these designs, discard ideas and refine interactions. Our approach was developed using web technology (HTML, CSS, Javascript), as well as services and libraries such as JQuery, D3.js and Google Material.

**Figure 2:**
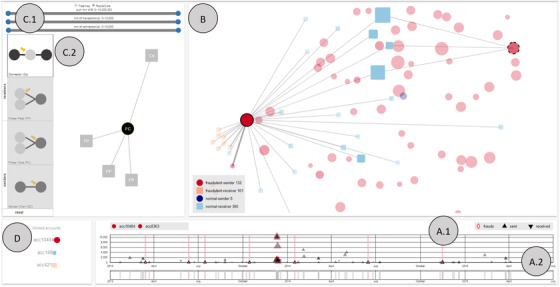
Screenshot of NEVA (Network dEtection with Visual Analytics). (A.1, A.2) Temporal Views: the two views present temporal detail (A.1) and overview (A.2) information. (B) Node‐Link View: this view represents the network of the analysed bank accounts. (C.1, C.2) Guidance‐Enriched Pattern Search: In this panel, we allow filtering the accounts with respect to the connections between them by using sliders (C.1) and changing the layout of the network through radio buttons (C.1). Moreover, we provide a guidance‐enriched network pattern specification, detection and filtering approach (C.2). (D) This view presents the history of inspected nodes for keeping track. In all views, elements that represent suspicious data are highlighted in red.

NEVA is composed of four main coordinated views that display different aspects of the data (see Figure 2). All views are connected interactively. We opted for well‐known visualizations in order to keep the learning curve as low as possible, and thus, to foster acceptance. However, all visualization techniques were chosen with respect to their suitability for the data and the tasks at hand [Mac86]. Next, we discuss the motivation of the different visual design choices made in our approach referring to A.1, A.2, B, C.1, C.2 and D from Figure 2.

4.4.1

##### A.1, A.2: Temporal Views

**Figure 3:**
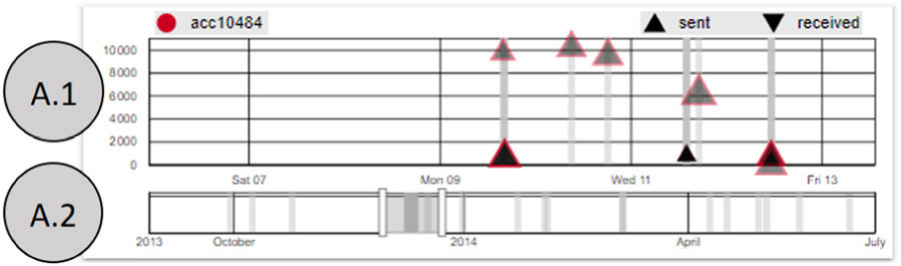
The two temporal views A.1 (detail) and A.2 (overview). The selected account is displayed at the top left corner, while the glyph legends are displayed on the top right corner. The investigators can also interact with the legend to filter the view.

The combination of both views (see Figure 3) supports a detail and overview visual analysis of time‐oriented aspects of the data [CKB09]. With the main focus of representing an overview, the view A.2 is smaller and supports brushing and filtering, which are then reflected in the detail view (A.1). By brushing A.2, the user zoom in the corresponding time gap in A.1 and, thus, potential overplots in the time representation (A.1) are spaced and clarified. In both views, rectangles highlight points in time (represented by the x‐axis) at which a transaction happened. These rectangles present a colour opacity feature that when accumulated over each other is decreased and, thus, facilitates the observance of overplots. The two views combination allows for analysing temporal details while preserving context information.

We opted for a 1D representation in the ‘overview’ view A.2 in order to keep it simple. This view serves two main functions, (1) giving an overview of all events of the data set and (2) allowing to zoom in and zoom out of the detail view A.1 with a brush interaction. Due to that, we constructed this view with just enough space on the y‐axis for brushing and filtering at an overview level.

The ‘detail’ view A.1 was designed to allow for analysing temporal aspects of transactions, represented by triangles. While the orientation of the triangles represents if a transaction was sent (triangle up) or received (triangle down), the amount of money sent is represented by its position on the y‐axis. The triangle's size encodes the suspiciousness score of the transaction and a red stroke indicates if a transaction's score is above a given threshold. Hovering the triangles provides detailed information about the transactions. For representing multiple transactions, we opted to present a glyph‐based approach in this view to better represent the collection of sent and received transactions. We sort all transactions before plotting them, from highest to lowest amount of money. Thus, small transactions, which result in small glyphs, are always plotted in the last layer. We tried bar charts but, when zooming out, one bar would need to aggregate multiple transactions (losing information, as line charts would do too) or to be very thin (losing visual appeal and adding difficulty to interactivity).

##### B: Node–Link View

We use visual variables to encode several features in the node–link diagram. Link width encodes the number of transactions between two nodes. Node size encodes how many connections (i.e. transactions from or to other accounts) a certain node has. In our scenario, the accounts that are investigated by means of this node–link visualization are filtered to those accounts that have been flagged as suspicious and other accounts that are connected to these suspicious accounts. Thus, the more connections a node has within this subset of possibly fraudulent accounts, the more suspicious it is itself. Thus, we use size to make them visible and to highlight big players. We used node colour to represent four categories of accounts: orange nodes represent suspicious receiver accounts, red nodes represent suspicious sender accounts, light blue nodes are receivers that are non‐suspicious so far and dark blue nodes are non‐suspicious senders. Since one of the main interest of the investigators is to distinguish (1) ‘senders’ accounts from (2) ‘receivers’ accounts, to assure a good differentiation of sender and receiver nodes, which are already differentiated by dark and light colour shades, we decided for double encoding these also to node shape: circles (senders) and squares (receivers). Node transparency represents how many suspicious transactions an account has compared to its total amount of transactions. The more suspicious transactions are associated with an account, the less transparent we draw the corresponding node. Node stroke is used during interactions to highlight selected or hovered nodes. Interactionwise, the investigator can select nodes, drag the camera view and zoom in and out of the view. When a node is selected, the view shows all first layer nodes with links and all second layer nodes without its links. Selecting second layer nodes for comparison is also supported.

**Figure 4:**
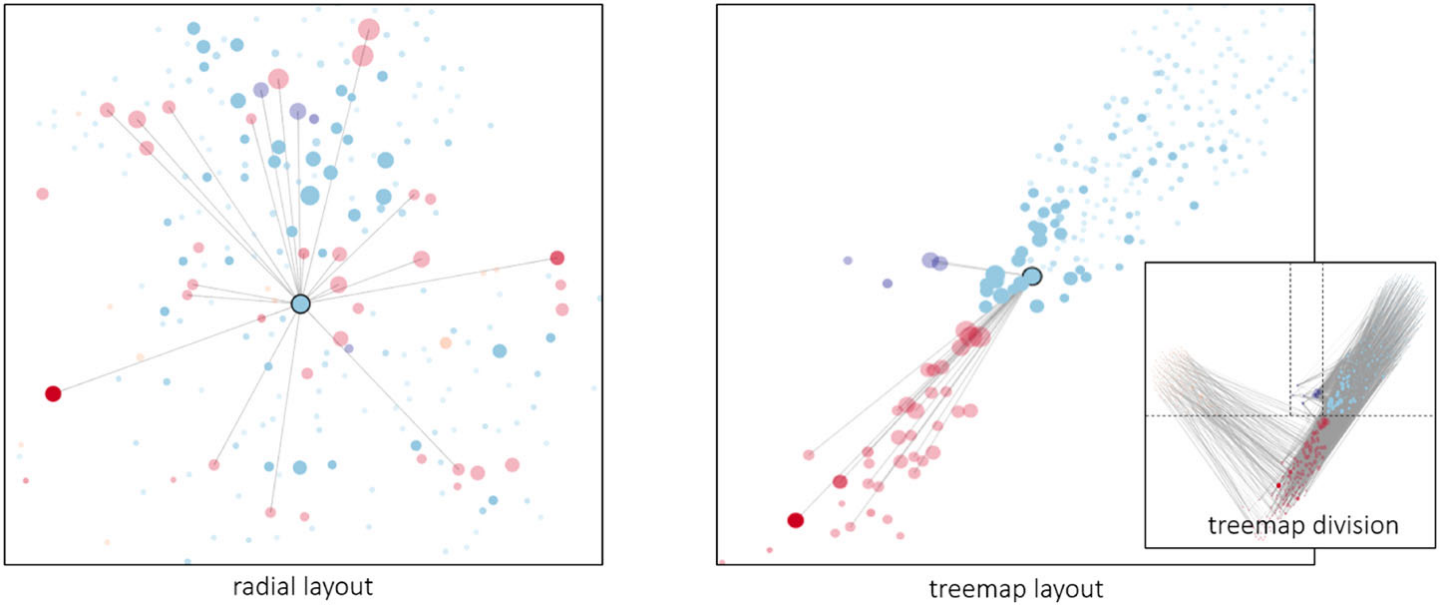
Two layout options presented by NEVA. On the left‐hand side, a radial layout which is better suitable for path‐finding tasks due to the distribution of nodes on the available space. On the right‐hand side, a treemap layout which groups the different types of accounts (fraudulent‐senders, fraudulent‐receivers, normal‐sender, normal‐receiver) into an underlying treemap segmentation of the available space.

We included two different force directed layouts: a treemap layout and a radial layout with cores (see Figure 4). A treemap‐like subdivision of the space with four divisions that represent the amount of each account type (fraudulent receiver, fraudulent sender, normal receiver and normal sender) and pulls these node types towards the centre of the respective subdivision. While creating attraction centres at each region, the nodes also get influenced by node–link spring forces. The influence of the spring forces avoid that the nodes merge and clutter to the same region centre points, even when they are being pulled in the same direction. The radial layout with cores assumes a light gravitational force in the middle in order to keep the elements close but the main aspect of it is the repelling force that the nodes apply to each other. By coupling the repelling force from all nodes with the link attraction force, we could obtain a network representation that visually separates subgraphs of related events. Both layouts pre‐calculate the position of nodes and do not allow drag and drop interactions in order to preserve the position of nodes, and thus, the mental map of the user.

To represent the network connections, we decided for a node–link view over a matrix view, since a node–link presentation better supports path‐finding [OJK18]. This feature facilitates the reasoning about circular schemes such as money laundering. While the position of node elements for different layouts (which all highlight different aspects of the network) presents an additional challenge, node–link diagrams are better suited for reading information from small and sparse graphs [KEC06]. In addition, a matrix view would introduce challenges of sorting and positioning accounts so that relevant accounts are close to each other to enable comparison, which usually demands several sorts of interaction features. Another beneficial feature of node–link diagrams is that they enhance reasoning about indirect paths between two and more nodes [KEC06]. Moreover, a node–link representation provides several visual attributes that can be used to support effective visual analysis without resulting in a confusing visualization (i.e. link width, node size, node colour, node transparency or node stroke). Furthermore, we provide a subgraph ‘drawing’ feature (i.e. drawing node–link connections) for querying the data set (see D: Guidance‐Enriched Pattern Search) which is a more intuitive approach than asking the investigator to draw a subgraph matrix.

##### C.1, C.2: Filters and Guidance‐Enriched Pattern Search

**Figure 5:**
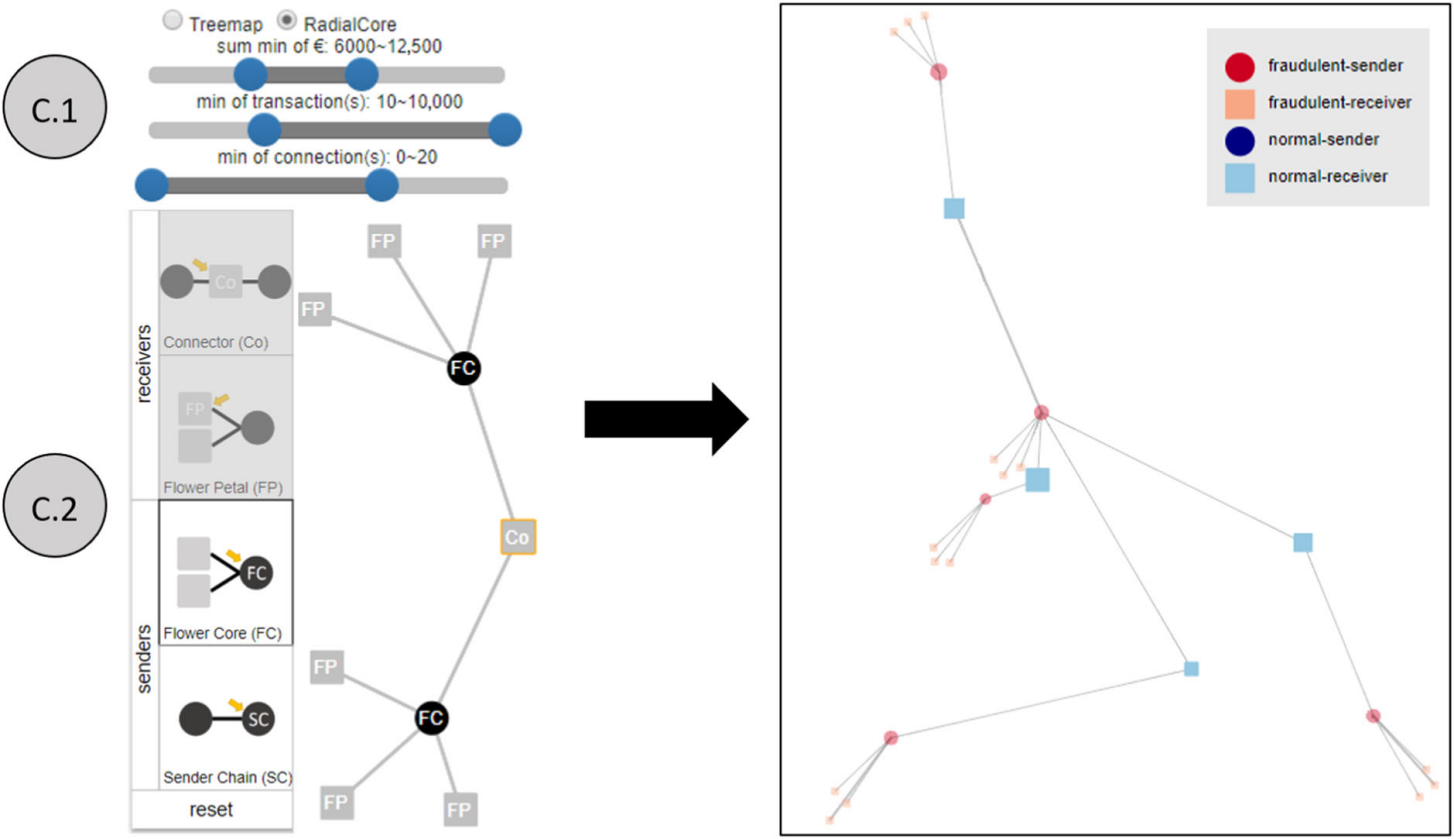
C.2 shows four possible subnetwork structures to be selected. Connectors (Co) are receiver nodes that are in between sender nodes. Sender Chains (SC) are sender nodes that are connected to each other. Flower Core (FC) is a sender node which is just connected to receiver nodes. Flower Petal (FP) are receiver nodes that are only connected to one sender node. Overall, this is an example of possible patterns that can be drawn and queried. C.1 provides sliders to apply interval filters. In C.2, the investigator drew a network pattern to query for: the black nodes present two FC connected to three FP each. The investigator then connected the two FC by a Co node. At the right‐hand side, we see the query result of the applied filters and the drawn pattern. Light blue nodes are Cos, dark blue nodes are SCs, red nodes are FCs and orange nodes are FPs.

In this panel, we provide filtering relations between accounts with respect to different features by using sliders (C.1), changing the graph layout through radio boxes (C.1) and a guidance‐enriched pattern generation, detection and filtering canvas (C.2). Based on the most common queries manually executed by investigators, we designed three sliders that can be combined to narrow down the content of an investigation (see Figure 5C.1). These sliders are used to filter network features (i.e. frequency of transactions and amount of money involved) and to specify filter intervals for these network features (min/max). Despite C.1 presenting a simple but very useful filter functionality, C.2 presents a more complex approach that is explained in Section 4.4.

##### D: Selection History

This view helps to keep track of the network investigations that have been performed. Through this view, we also can reselect already investigated nodes.

### 4.5 Node–link patterns & guidance‐enriched pattern canvas

Our approach to provide guidance for the detection of interesting patterns within the network was motivated by the work presented by Cheng *et al*. (see figure 15 in [CNY*17]). In their work, the authors elaborate that different combinations of only four nodes generate nearly 200 different types of subgraphs, however, just 20 of these subgraphs could be found in the data set. Thus, as the number of different possible combinations of *n* nodes increases exponentially, we guide users in constructing subgraphs that are used to query the data set. Showing them which combinations of patterns make sense because they are actually present in the data considerably reduces the number of possible subgraphs.

Besides simplifying pattern searches by allowing the user to simply draw the subgraph that should be queried, we also facilitate these subgraph query constructions with guidance. Guidance is seen as a computer‐assisted process that gradually narrows the knowledge gap that hinders effective continuation of the data exploration and analysis. It provides prospective assistance so that users can make sense of the data on their own [CGM19, CGM*17, CGM*18]. In analogy with the auto‐complete text guidance assistant in VISAGE [PTE*16], we propose a solution that preserves the investigator's mental map. Common text auto‐complete features might disrupt an investigator's mental map [PHG06, APP11] by suggesting well‐known or most used queries. Instead of influencing the query with our guidance system, we apply guidance only to avoid that queries will produce empty search results. Thus, we stepwise guide the user through all possibilities of subgraph constructions that actually exist in the customer graph. Node combinations that do not exist in the data, cannot be drawn; only the ones that exist are available. Moreover, our visual query approach is more user‐friendly than a text‐based approach that might require scripting knowledge. Based on Ceneda *et al*.'s definition of different guidance degrees [CGM*17, CGM*18], we categorize the guidance degree provided in view C.2 as: ‘directing guidance’, since it narrows down the multitude of options which in theory are possible at each step of constructing a subgraph, to those options that actually make sense. The input is network data as well as the user's actions (i.e. which node is currently selected) and the output are possible additions to the drawn subgraph, which is used to query the data. In order to perform a guidance‐enriched network pattern exploration and search function (see Section 4.1), first, we defined four different categories of nodes according to their relations [i.e. ‘Connector (Co)’, ‘Flower Petal (FP)’, ‘Flower Core (FC)’ and ‘Sender Chain (SC)']. All four categories are described in Figure 5. Reducing a pattern representation to these categories of linked nodes proved to be very powerful by not only being able to represent all patterns described in Cheng *et al*.'s approach [CNY*17] but also for being able to construct any money flow subgraph existing in our real‐world data set. The complete representation is guaranteed for our use case because each node from our data set falls into one of the four classes. Thus, we use these node categories as a base for building the pattern search feature in view C.2. When it comes to performance, all subgraph queries applied to our real‐world data set (see Section 4.2) during development and evaluation of NEVA, presented ‘instant’ results (i.e. execution average of 3 ms when using a computer with Intel Core i5‐2520 2.50 GHz with 8 GB RAM).

In view C.2, the investigator can draw any subgraph by adding and linking the previously proposed node–link patterns. When drawing such a subgraph, NEVA guides the investigator by providing possible links to add to the subgraph, while avoiding non‐existing constructs or links that make no sense. The options are automatically calculated based on the node that the investigator selects in the query canvas, and thus, the provided guidance is an active answer on the user's action and his/her knowledge gap (i.e. complete awareness of which patterns exist in the customer graph). In this way, our guidance support facilitates pattern generation, detection and filtering tasks [CGM*17, CGM*18]. Therefore, we name it Guidance‐Enriched Pattern Canvas.

Our guidance approach prevents two different types of common errors when constructing subgraphs: First, if a structure cannot be found in the current data set, it is not possible to draw this structure in the Guidance‐Enriched Pattern Canvas. For instance, let us assume that the maximum connections of any FC node in a data set are three FP nodes. After drawing the third link from an FC node to an FP node in the drawing area of view C.2, the FP node selection option would be greyed out. The second type of error prevented by our guidance system is the combination of patterns and nodes that do not make sense (i.e. the logical structure and connection of different types of nodes). For instance, two FP nodes cannot be connected to each other, otherwise it would represent an SC node (SC). On the other hand, at least two SC nodes must be connected to each other. For each added node in the drawing area, the query is immediately executed and all other views are updated accordingly. With that in mind, we illustrate a real‐world example in Figure 5. C.2 shows that a Co node is selected (see the yellow bordered squared node) to extend the query pattern. Since a Co node cannot be linked to another Co node and neither to an FP node, these options are greyed out.

##### Subgraph search algorithm

We first categorize all nodes in the data set by categories (FP, FC, SC or Co). Once the nodes are categorized, we can search for matching subgraphs in the data model. We define $G_{t}=\left(V_{t},E_{t}\right)$ as our data model graph, being $V_{t}$ the vertex set and $E_{t}$ the set of ordered pairs of nodes representing the graph edges. We also define $|V_{t}|=n$. The subgraph pattern that the user constructs during a query is defined as $G_{p}=\left(V_{p},E_{p}\right)$. Next, we use our pattern matching algorithm to look for a subgraph $G_{r}=\left(V_{r}\subseteq V_{t},E_{r}\subseteq E_{t}\right)$. Considering that the $G_{p}$ nodes represent different categories, which identify a set of nodes in $V_{t}$, this algorithm conducts the following operations: (1) for every node category $v_{p}\in V_{p}$, it extracts the nodes $v_{t}\in V_{t}$ that match the constraints defined in $v_{p}$ and assigns them to $V_{r}$; (2) for every node now in $V_{r}$, it checks whether its edges connect to $E_{r}$. An edge $e_{t}=\left(u_{t},v_{t}\right)\in E_{t}$ belongs to $E_{r}$ if $u_{t},v_{t}\in V_{r}$ and they were extracted from the two different categories nodes in $V_{p}$ connected by an edge $e_{p}\in E_{p}$.

The algorithm has a quadratic asymptotic time complexity $O(n^{2})$. The first step (1) has to be repeated for each node in $V_{p}$, therefore it has a complexity of $O(|V_{p}|)$. We can perform the second step (2) in O(d) time for each node, being d the out‐degree of a node. Therefore, considering that from $V_{p}$ we extract the nodes to insert in $V_{r}$, the time complexity of the algorithm is $O(|V_{r}|d)$: $|V_{r}|$ is at most n (when the queried subgraph includes all the nodes in $V_{t}$) and d is at most n−1 (when $v_{r}$ shares an edge with all the other vertices in the graph).

However, in practice, we could find that also with very dense graphs and real‐world data our implementation was able to find subgraphs with an efficient time, enabling a real‐time interactive exploration. Even if the general pattern matching problem is NP‐Hard, in our case the problem is greatly simplified by the precalculated categories that maps the nodes in the target graph.

## 5 Solving Real‐World Tasks with NEVA

In this section, we aim to present four real‐world insights about false‐negative and false‐positive cases that were confirmed by investigators using our approach.

### 5.1 False‐negative alarms

**Figure 6:**
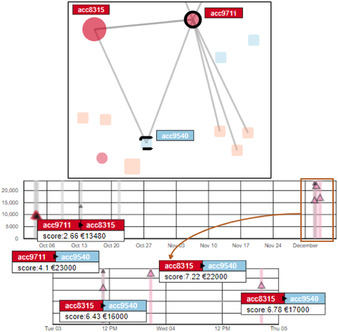
The triangle relationship between three accounts, i.e. the flagged accounts acc8315 and acc9711 as well as acc9540, an initially non‐flagged account. It is possible to observe high‐value transactions between the two flagged accounts and in addition, from both accounts to the same target account (acc9540). This indicates that acc9540 might be a fraudulent ‘straw person’.

When inspecting the ‘acc9711’, one of the first network features that appeared to the investigator is its connection with another sender account, which would present an SC (see Figure 5). After a quick inspection of the linked fraudulent account ‘acc8315’, the node–link view reveals that both accounts have the non‐flagged account ‘acc9540’ as a common receiver (see Figure 6). By observing the multiple transactions involving a high amount of money from the two connected fraudulent accounts, we conclude that the non‐flagged account ‘acc9540’ should also be considered suspicious in case of a fraudulent network scheme.

**Figure 7:**
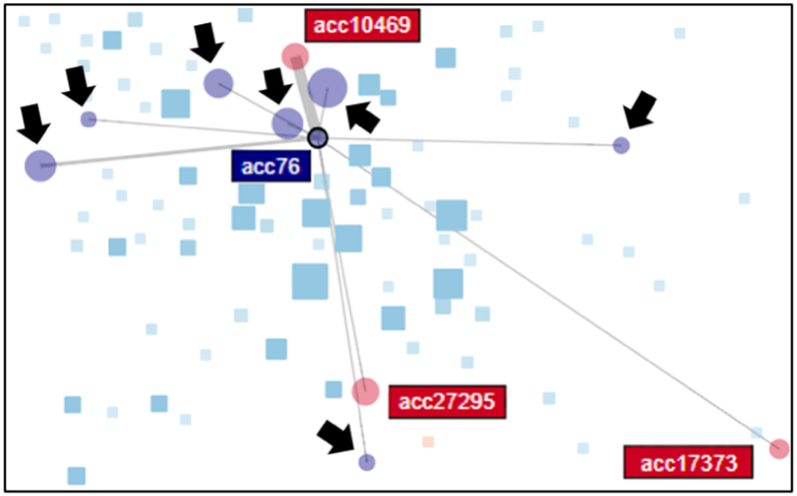
A case that acc76, a non‐flagged sender, is detected as a ‘man‐in‐the‐middle’ node that connects three flagged senders. Moreover, it sent money to seven additional non‐flagged accounts. Thus, NEVA automatically includes these non‐flagged accounts for a comprehensive investigation of possible fraudulent networks (see Section 4.2).

Selecting the ‘man‐in‐the‐middle’ (i.e. the non‐flagged sender account ‘acc76') revealed that it received money from three different fraudulent senders (see red nodes in Figure 7). Since this account has suspicious connections and is also a sender account, the ‘man‐in‐the‐middle’ search algorithm was also applied to its connections. This revealed seven additional non‐flagged senders that have common receptors with flagged accounts (see arrows in Figure 7). Further investigation confirmed the involvement of those accounts in a larger fraudulent network scheme. In this case, the ‘man‐in‐the‐middle’ selection highlighted a potentially fraudulent network of accounts that were not flagged as suspicious by the automatic detection mechanism.

### 5.2 False‐positive alarms

The account ‘acc4238'presents two flagged money transactions on the same day to two different accounts. First, a 6500€ transaction to account ‘ib3614’ that resulted in a score of 25, and second, a 50€ transaction to account ‘ib3613’ that resulted in a score of nine. While the first transaction is worrisome, especially due to the high amount of money involved, the second transaction involves just a small amount of money. Although the second transaction resulted in a much lower score than the first one, it still exceeded the threshold of the automatic detection algorithm. This sensitive behaviour might be a result of the first alarm (from the same day) and can be neglected. Thus, this case presents a false‐positive alarm that would not be easily detected and justified without data exploration motivated by a VA tool.

**Figure 8:**
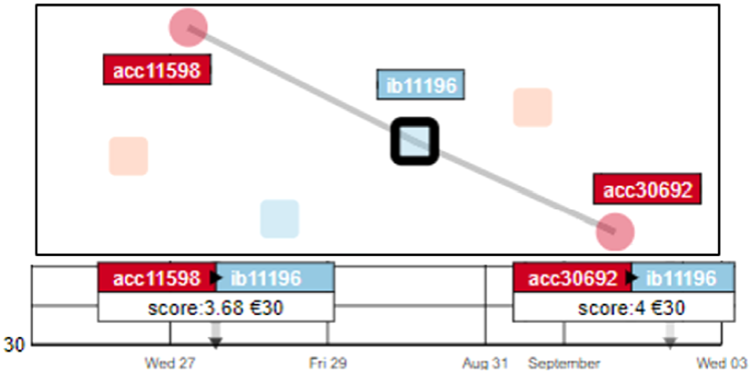
The selection analysis of the ‘man‐in‐the‐middle’ account ‘ib11196’. Although this account is connected to two fraudulent senders, the temporal view reveals a non‐suspicious behaviour of only two received transactions of 30 € , which makes it very unlikely that this account is involved in any fraud.

While ‘man‐in‐the‐middle’ nodes are not flagged by the automatic detection mechanism, investigators still consider them as highly suspicious and used NEVA to further invest them individually. If an investigator analyses such a suspicious case and comes to the conclusion that it is harmless, it can be considered a false‐positive alarm. In Figure 8, we present a case with a non‐flagged node (‘ib11196') being the ‘man‐in‐the‐middle’ of two different fraudulent accounts (‘acc11598’ and ‘acc30692'). The two transactions involved show a small amount of money (30€ each). Although frequently sending small amounts of money could indicate a certain type of fraud, NEVA shows that those two relations do not have any previous or subsequent involvement. Thus, it does not present a critical account.

## 6 Evaluation

In order to estimate the usability of our approach, we conducted a qualitative evaluation with six domain experts. By asking them to answer three research questions (RQ), a qualitative study allowed us to get the investigators feedback and reasoning about the insights that were gained while using NEVA.

  
**RQ1: Comparison**. What are the advantages and disadvantages of NEVA compared to the tools which investigators usually use?
  
**RQ2: Insights**. What kind of insights can be generated with NEVA?
  
**RQ3: Improvements**. Do the investigator miss any features or have suggestions for improvement?

6.1

6.1.1

##### Participants, Data set and Tasks

We recruited six potential investigators from our collaborating bank who had never seen the prototype before. Qualitative studies are a useful means to generate insights with relatively few study participants [IIC*13, KP15]. All participants had previous experience with visualizations for data presentation.

During the evaluation, we used an anonymized real‐world data set from our collaborators covering an interval from January 2013 to April 2015. After the data setup (see Section 4.2), 661 different accounts with a total of 1 527 583 transactions of different types (i.e. net banking transactions and automatic payments of bills) were selected to be inspected. These tasks are structured according to the analytical task taxonomy by Andrienko and Andrienko [AA06], distinguishing between elementary and synoptic tasks.

To evaluate the task performance of our approach, we stipulate four tasks directly related to the proposed requirements (see Section 4.1). Task 1 is designed to evaluate R1, Task 2 is designed to evaluate R2 and so on.

  
**Task 1**: Identify false‐negative alarms for any period of time.
  
**Task 2**: Identify false‐positive alarms for any period of time.
  
**Task 3**: Find at least two types of potential frauds.
  
**Task 4**: Query any fraudulent pattern known to you.

Our evaluation design covers the highlighted problem complexities (see numerical references of Section 3.2): Tasks 3 and 4 are addressing context (2) and fraud classification (4) challenges, all tasks are addressing time‐oriented data analysis (3), and scalability (1) is put to the test by the usage of a real‐world data set with 77 000 accounts (see Section 4.2).

##### Procedure and Collected Data Analysis

The study procedure took place in two locations, a meeting room at the university and a meeting room at the bank headquarters. The participant and the evaluation moderator were present. In addition, audio and video recording software were used to support further data analysis. First, a short introduction to the study's goal and the meeting schedule were presented. Next, a semi‐structured interview took place in order to better understand the current approach for network FFD and the participant's VA background. Then, the participant was invited to perform the proposed tasks using NEVA. While interacting with the prototype, the participant was encouraged to think aloud (RQ2). After this phase, another semi‐structured interview was conducted in order to collect feedback (RQ1, RQ3).

After the evaluation session, we analysed the collected qualitative data (notes, audio and video recordings). In order to understand how effective our approach was supporting exploration and sensemaking (RQ2), we opted to use Klein‘s model (see also [KMH06a, KMH06b]) to interpret the collected data due to its broad [PSM12] intelligence analysis approach. For this reason, we adopted three categories from Klein [Kle13] to categorize notes, audio and video recordings.

##### Connection

These are the resulted insights of the combination of two or more views. For example, two views showing different aspects of the same data elements.

##### Coincidence

These insights are results of events that in a first moment do not present obvious connections but through the viewers’ eyes seem to have a relationship. For example, two data elements with a similar outlier position might have the same non‐identified source.

##### Curiosity

These are motivation insights. For example, seeing one data element positioned in a different place when compared to the others, arouses the viewers’ interest in finding an explanation to that.

### 6.2 Results

All the participants were able to achieve satisfactory results on the four tasks with an average duration of 12 min. The first interview session (before trying the prototype) took about 15 min and the second interview (after trying the prototype) took about 25 min.

In this subsection, we present the results from the evaluation session with respect to our RQs.

#### 6.2.1 RQ1: Comparison

The participants usually use visualization approaches for data analysis and presentation purposes. Some of the tools cited by the participants were: Microsoft Excel [Mica], Microsoft PowerPoint [Micb] and Tableau [tab]. From the available visualizations of the mentioned tools, they are most familiar with line charts, pie charts and bar charts.

When comparing our approach to the other tools, some participants highlighted that interactivity of our approach was helping a lot with the filtering of ‘interesting elements’ and the analysis of relations. Concerning the system precision on representing and filtering, one participant commented: ‘just by looking at the graphs and interacting with it, I can detect a small number of suspicious nodes and gaining an initial understanding of them’. Another participant commented: ‘The interactive technique is not only good for detecting suspicious patterns but also to exclude false alarms’. Besides that, many aspects of our approach such as the design choices of the node–link diagram and the temporal view, the history track panel, the slider filters and mainly the network pattern drawing tool were very well received. The participants also demonstrated an interest in showing our solution to business, meaning that a potential official tool could be created based on our prototype. With that and other motivating statements, we reason that NEVA is a positive improvement to the current workflow of FFD investigators.

#### 6.2.2 RQ2: Insights

**Figure 9:**
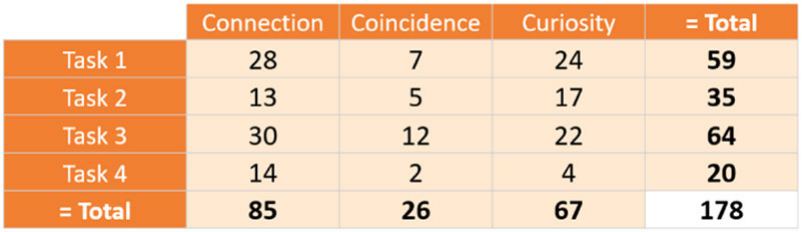
Insight summary. The appearance and the sum of each insight type with respect to the tasks performed.

Three kinds of insights were counted during the evaluation session: Connection, Coincidence and Curiosity. We present the distribution of a total of 178 identified insights (average of 29 for each participant) according to the performed tasks in Figure 9. **Connection**. The dominant appearance (47.75%) of this insight is mainly due to the multiple coordinated views of our approach which link the node–link view (B), the temporal views (A.1, A.2) and the Guidance‐Enriched Pattern Search (C.1, C.2). For example, observing a node in the node–link view would lead the investigator to select it and analyse its transactions in the temporal view. Another example is that investigators constantly checked changes in the node–link view when drawing a pattern with the network pattern drawing tool. **Coincidence**. With the smallest appearance (14.60%), this insight predominantly occurred during Task 3. Since this task was about investigating different types of frauds, it is plausible that this insight type is about finding hidden relations, played a main role. **Curiosity**. During the investigation of false‐negative cases (Task 1), this was the main insight type with six appearances. By interpreting suspicious transactions (i.e. links) in the node–link view, the investigators often inspected also the nodes to check if the transactions were really suspicious. This represented (37.64%) of the total insights.

#### 6.2.3 RQ3: Improvements

During the last part of the evaluation sessions, the participants were encouraged to make suggestions about how the approach could be improved from their point of view. The first suggested feature was to allow excluding nodes and transactions in case the investigator finds false‐positive alarms. Another feature suggested was an ‘undo’ button that would return the whole system to how it was one action before. A mouse‐over window for hovering edges, giving an overview of the frequency of transactions between two nodes before inspecting it in more detail in the temporal views was also desired. Concerning the temporal views, a participant recommended to add a selection box that would allow the investigator to observe only the interaction between the two selected nodes instead of only highlighting them visually. We consider all other suggested improvements as minor usability issues that will be added in the next iteration of our approach.

## 7 Discussion

In this section, we discuss (1) how our approach fulfills the previously defined requirements, (2) benefits of a potential integration with EVA [LGM*18] and (3) limitations of our approach as well as future research challenges in this field.

### 7.1 Requirements

We designed and developed NEVA to support a guided exploration and informed reasoning about fraudulent networks in the field of FFD. Our solution provides different levels of data abstraction. Moreover, it supports important tasks in the context of FFD, such as (1) inspecting relations between bank accounts, (2) analysing temporal aspects of financial transactions with detail and overview information, (3) filtering the network of accounts with respect to the characteristics of relations between accounts, (4) querying for specific network patterns supported by novel guidance mechanisms and (5) identifying straw persons and 'humans‐in‐between’ by ranking all accounts connected to a selected account in two layers: direct connections (first‐level connections) and all connections of these first‐level connections (second layer connections). Moreover, all views are connected, i.e. they consistently reflect all changes applied to the data (i.e. filtering, selections, modifications).

Investigators can analyse temporal aspects using an overview and detail approach of the temporal views A.1 and A.2 (Figure 2). Since it is very difficult to encode all relevant data aspects in one view, these temporal views allow for the detailed inspection of transactions while preserving the bigger picture. Another feature that supports reasoning about the temporal aspects of transactions is the filtering consistency between the views. The combination of these views helps to reason about the temporal sequence and consistency of sent and received transactions of a selected account. Moreover, our approach also enables direct comparison of the transaction histories of two accounts. This feature supports the investigator in reasoning about a potential fraudulent collaboration between two or more accounts.

Since we used a real‐world data set for development and evaluation, our approach scales to the scope of data sets typically used for real‐world FFD tasks. Due to local law restrictions, bank institutions are allowed to keep records for a maximum period of 7 years. Thus, our approach scales well even to the highest possible number of transactions existing in available data sets (32,175 transactions).

The node–link view allows for an analysis of connections between flagged and non‐flagged accounts. After identifying suspicious relations, by using the temporal view, we can reveal transactions with suspicious features and/or to suspicious accounts. This enables investigators to investigate these accounts and make better informed decisions about the suspiciousness of these accounts, and thus, to reveal potentially false‐negative cases **R1**. Similar inspections can be performed the other way around, starting from accounts flagged as suspicious and investigating the reasons for which this account was flagged by the automatic detection mechanism. For example, during the evaluation session (Task 2), one investigator filtered the data displayed to a low amount of money transactions (using view D) and quickly inspected the remaining accounts. After a few minutes, s/he could already confirm that a good part of those accounts presented false‐positive alarms **R2**.

The identification of different types of fraudulent behaviour **R3** is a very difficult and sensitive task that requires the combination of all views of NEVA. The differences of account relations become more evident during visual analysis. In addition, the understanding and identification of fraud patterns might be registered for further usage during a network pattern query **R4**. We also presented a tool (view D) to guide the search for relational patterns that might support investigators in formalizing queries and also in the categorization of fraud types.

### 7.2 Potential integration with EVA [LGM*18]

Since we already designed and developed an FFD tool focused on different techniques, we strongly believe that NEVA would lend itself to be integrated with this approach. NEVA adds new features for network analysis and tools to the investigation process. Using interactions such as node selection, the investigator could link the two approaches in order to perform different analysis without losing the investigation track. Thus, as an extension, the best of both approaches would sum up in a better and more complete solution.

### 7.3 Limitations & further work

Network pattern analysis and outlier detection are constant research challenges. Additional layers of complexity should be considered when involving time‐oriented and multi‐variate data. Based on current limitations, we derive research challenges to inspire future works.

7.3.1

##### New Accounts Monitoring

Since our automatic algorithm is based on scores which are calculated based on the account's history, we cannot efficiently evaluate newcomer accounts. Moreover, it is known that some frauds involve completely new accounts in order to hide from these algorithms. However, for those frauds other algorithms apply. That being said, we strongly believe that FFD could benefit from solutions for (1) analysing first steps of new accounts individually and (2) keeping track of accounts that cannot be interpreted by a history‐based system. Another possible challenging future work would be (3) to support the migration of new accounts analysis for a score‐based approach.

##### Subgraph Search Limitations

The Guidance‐Enriched Pattern Search (view C.2) provides a more intuitive search and better understanding of the data through its interactive and responsive exploration. However, this (i.e. the subgraph isomorphism problem) is an NP‐complete problem. For the analysis of larger data sets, the usability of this technique depends on the available computation power and/or advances in the state of the art of subgraph matching algorithms. However, as we did in NEVA, the performance of the query algorithm can be improved when the attributes of nodes are used to constrain searches.

##### Temporal Analysis of Multiple Accounts

NEVA's temporal views (A.1 and A.2) already support the temporal analysis and inspection of transactions. With these views, we provide support for comparing two accounts. In future work, it would be interesting to include a temporal view that supports the analysis of all accounts of the selected network in a non‐cluttered way.

##### Collaborative Investigation

Some analysis scenarios, such as a governmental investigations, can be at huge scale and involve many investigators over a grand period of time. Many countries recently faced cases of political corruption involving illegal money transactions that take years of analysis. Other examples of huge investigations are Wiki Leaks [BHM13] and analysis of cryptocurrency networks. These types of investigations might demand for a collaborative analysis. Distributing workload and double checking hypotheses would potentially present faster and more objective results.

## 8 Conclusion

Based on our experience in this field and in tight collaboration with domain experts from a national bank, we iteratively designed our VA solution for FFD network analysis, called NEVA. Our approach follows the VA principles of intuitive and interactive visualizations in combination with analytical techniques. All design and interaction choices were made with special consideration of the previously defined requirements and with respect to the limited experience of FFD investigators with visual exploration tools. Our approach consists of an automatic computation of profile‐based suspiciousness scores for each transaction in combination with and interactive multiple‐coordinated view approach to explore and reason about the behaviour of bank accounts. By showing ‘data variation' [Rob07, WBWK00] NEVA improves the quality and speed of the investigation process.

We designed and evaluated NEVA with real‐world data and six real‐world domain experts, which helped to assess the required scalability of the approach. The added value of our approach is supported by (1) the analysis of insights gained with the help of NEVA during our evaluation session with real‐world investigators (see Section 6) and (2) the additional fraudulent cases identified with the help of NEVA that were later confirmed by investigators to constitute actual cases that demand for further investigation (see Section 5). The results of the evaluation session helped us to assess the positive impact of our approach in a real‐world setting. The improvement of task performance and the number of insights gained while using NEVA confirmed its usability and efficiency.

Based on our study results, we also propose possible future research directions that not only would be of added value to the FFD domain, but also to fields that share similar problems and data characteristics (i.e. multi‐variate, time‐oriented and network data aspects), such as biology, medicine, insurance companies or the public sector.

## Supporting information


**Data S1**
