# Supplementary material for: NEVA: Visual Analytics to Identify Fraudulent Networks
Source: Comput Graph Forum. 2020 Jun 19;39(6):344–59. doi: 10.1111/cgf.14042 (PMC7584106; doi:10.1111/cgf.14042)
Supplement: Supplementary file 1 — Data S1 [file CGF-39-344-s001.pdf]

“NEVA: Visual Analytics to Identify Fraudulent Networks”

video

https://drive.google.com/open?id=1WjM2QL0h-dDTUrSrkDDAped97znzgC_i
